# Untapped options to reduce waste from blister packaging for tablets and capsules

**DOI:** 10.1007/s00228-023-03594-1

**Published:** 2023-11-18

**Authors:** Olivia C. Falconnier-Williams, Walter Taeschner, Andreas Hille, Ariane D. Falconnier, Walter E. Haefeli

**Affiliations:** 1 Department of Clinical Pharmacology and Pharmacoepidemiology, Heidelberg University Hospital, Im Neuenheimer Feld 410, 69120 Heidelberg, Germany; 2 Frosch Apotheke, Basler Str. 19, 79539 Lörrach, Germany; 3 Drossapharm AG, Pharmaceuticals, Birsweg 1, 4144 Arlesheim, Switzerland; 4 4104 Oberwil, Switzerland

**Keywords:** Pharmaceutical packaging, Solid oral dosage forms, Generics, Blister, Packaging waste, Waste management, Germany

## Abstract

**Purpose:**

In Europe, most medicines are taken orally and primarily packaged as single solid oral dosage forms (SODF) in blister chambers (alveoli) arranged on blister cards. Blister cards are constructed as multilayer laminates of aluminum (Al) foils and/or various plastic polymers bonded together, forming the alveoli, which are separated by more or less large gaps. We calculated the amount of packaging material (and thus waste) generated annually for the packaging of the most commonly prescribed SODF in Germany and estimated how much waste could be saved by rearranging the alveoli.

**Methods:**

For this purpose, we analysed the SODF of the 50 most frequently prescribed medicines that were packaged in alveoli (*N* = 45; 13 of aluminum-aluminum blisters, 32 of mixed materials), measured and weighed their packaging material and content, calculated the annual amount of waste produced from them, and estimated how much waste could be saved if the alveoli were optimally positioned on the blister cards. In addition, we examined the variability of the blister packaging of eight groups of commonly prescribed generics of the same strength.

**Results:**

Detailed analysis of the blister cards revealed that most of the material (69%) was used for the space between blisters and that aluminum-aluminum alveoli were more than four times larger than the packaged SODF. The (conservatively) estimated annual amount of composite waste generated for the primary packaging of these SODF was 3868 t (and extrapolated to the entire German pharmaceutical market 8533 t), of which an optimized arrangement of the blister chambers, i.e., a 2-mm sealing area around each alveolus and the arrangement of the SODF in 2 rows, would save approximately 37%.

**Conclusion:**

Considering that other ecological strategies are not yet mature, the optimal arrangement of blister chambers would be a captivatingly simple and, above all, immediately implementable strategy to avoid large amounts of avoidable waste.

**Supplementary Information:**

The online version contains supplementary material available at 10.1007/s00228-023-03594-1.

## Introduction

In Germany and most European countries, the single-dose blister pack (alveolus) is the predominant packaging for solid oral dosage forms (SODF) of drugs such as tablets and capsules. As primary packaging, alveoli protect the SODF from adverse external influences (e.g., moisture, oxygen, light, biological contamination, or mechanical stress) [1], provide tamper evidence and protection, and can serve as a reminder package to improve patient adherence [2]. They must not alter the ingredients of the drug and the drug must not be adsorbed to the package material [3, 4]. Alveoli usually consist of two aluminum foils or an aluminum and a plastic foil or plastic multilayer laminates joined together and welded with adhesive; therefore, they consist of composite materials that are difficult to separate and reuse [5, 6]. For this reason, and because the medications taken, as well as any not taken, end up in waste, all blister cards are currently disposable materials and their volume should be minimized as much as possible.

Climate change is considered the greatest current global health threat [7], and in countries of the Organisation for Economic Co-operation and Development (OECD), China, and India, the health care system itself contributes approximately 5% of total greenhouse gas emissions [8]. In the United States of America (USA), this figure continues to rise and is currently estimated at 8.5% [9]. The majority (~ 80%) of the healthcare sector’s carbon emissions come from the supply chain of its services and goods, i.e., indirect greenhouse gas emissions (Scope 3), which includes blister packaging.

Germany is Europe’s largest pharmaceutical market with an annual revenue of EUR 61.4 billion (bn), corresponding to 1.5 bn medication package units distributed in 2020 [10]. The vast majority of all drugs taken by patients are SODF, 85% of which are packaged in alveoli [1]. The weight of each alveolus (blister chamber) depends on the dimensions of the SODF to be packaged in it. In addition, the material used can influence how small an alveolus can be designed; for example, alveoli made of pure aluminum tend to be larger for technical reasons, because—unlike thermoforming of plastics—it is not possible to form aluminum at a 90° angle [5]. Because alveoli are usually arranged in several on a blister card, the material requirement also depends heavily on the distances between the blister chambers and their spatial arrangement. When designing the blister cards, two influences must be taken into account; on the one hand, the blister cards should be shaped in such a way that they can be easily handled by the target population (i.e., healthcare professional, patient, or caregiver). On the other hand, the existing blistering machines often restrict the free arrangement of the blister chambers.

If it is not (yet) possible to use environmentally friendly, emission-neutral packaging materials and design the packaging and its logistics in a sustainable way (ecodesign) [11], at least reducing the amount of waste in the primary packaging of pharmaceuticals would be an important first step. This will reduce the environmental impact associated with the production and distribution of the packaging material and also reduce pollution and cost for its unavoidable disposal.

The aim of this study was (i) to obtain a representative overview of the way SODF are packaged in Germany, an important pharmaceutical market, (ii) to calculate how much waste is generated by the primary packaging of oral medicines, and (iii) to make suggestions on how this amount of packaging can realistically be optimized and what savings could be achieved.

We assessed the impact of a key element of ecodesigning [11], the use of blisters [12], whose environmental impact is considered particularly variable and not optimized [13]. Our analyses of the 45 top-selling SODF in Germany have shown that a better spatial distribution of the alveoli on the currently marketed blister cards would consume at least 30% less composite material. Extrapolating these findings to the entire German SODF market resulted in a reduction in packaging waste of > 3000 t per year.

## Materials and methods

### Data source

#### Top-selling SODF

On the basis of the report of the Wissenschaftliches Institut der AOK (WidO) 2021 [14], we have determined the 50 drugs with the highest number in sales to patients with statutory health insurance in 2020. Three metered dose inhalers and two multidose containers were excluded, yielding 45 medicinal products of the 50 top-selling drugs whose primary packaging was an alveolus (Supplemental Table S1). Accordingly, the blister cards of these 45 brands provide the basis for all comparisons, extrapolations, and general statements made in this analysis.

#### Variability of generic products

Because the 50 top-selling brands consist of a range of different active ingredients and strengths, it did not seem meaningful to only compare the blister cards of these brands. Rather, it was also of interest to analyse how big the differences in packaging were between exchangeable brands (generics) of the same strength. For this purpose, eight off-patent (generic) active ingredients (acetylsalicylic acid, ibuprofen, metformin, omeprazole, pantoprazole, sertraline, tamsulosin hydrochloride, and valproic acid) were selected based on their availability in a large German community pharmacy (Frosch-Apotheke, Lörrach, Germany) where the data collection was carried out (convenience sample). This second sample included 44 brands, one of which was one of the 45 top-selling brands in blister packs and was therefore also included in the other sample. Together with the 45 top-selling blister-packed brands, this therefore resulted in a total of 88 blister cards analysed (Supplemental Table S2).

### Data acquisition

**Fig. 1:**
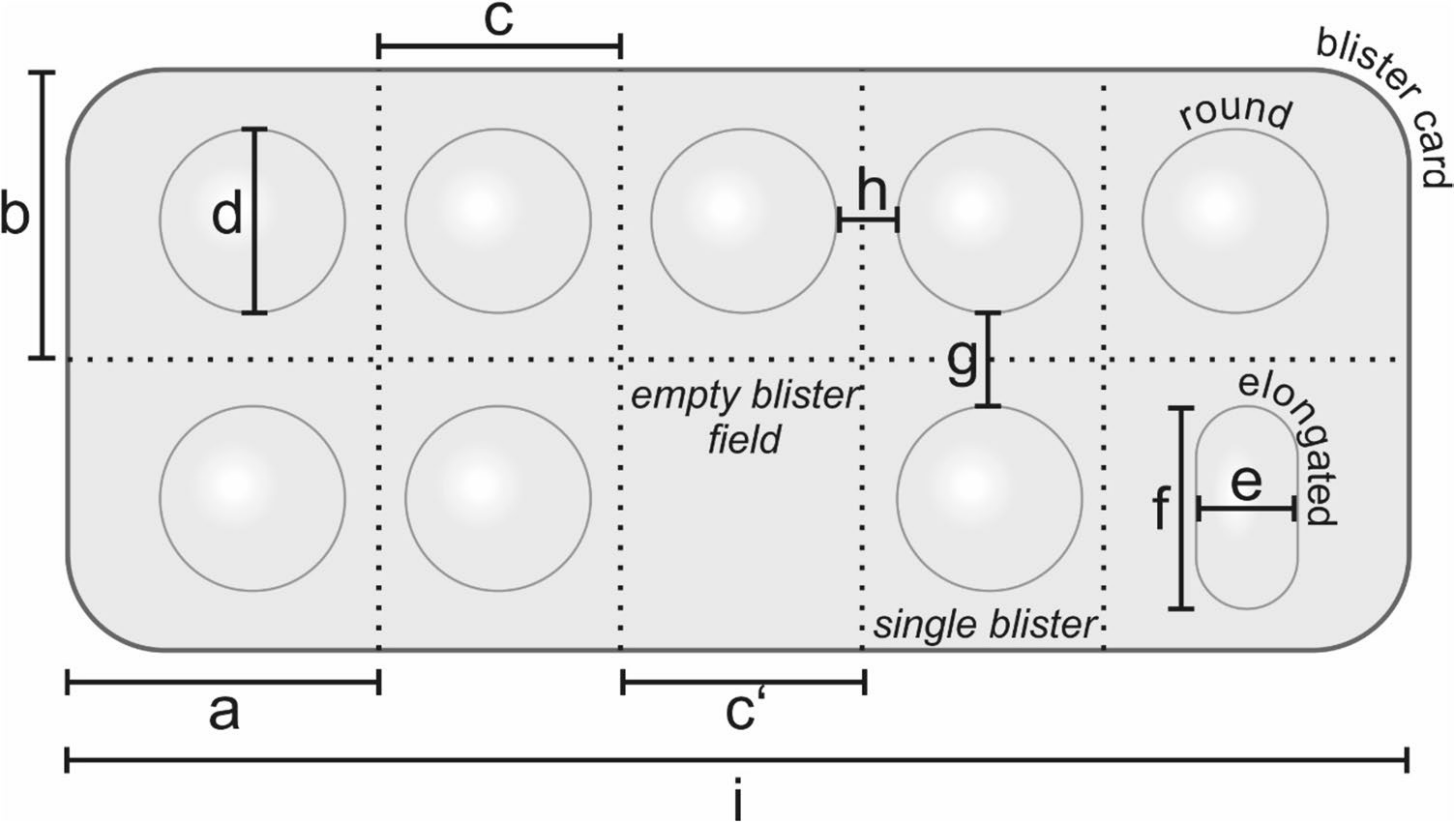
To assess blister dimensions and void space in blister packs (primary packaging) of a representative sample of solid oral dosage forms currently on the market in Germany, the characteristics and spatial dimensions of blister packs were assessed by recording the material of the blister cards (aluminum, plastic) and measuring the blister cards (a, b, c, i), the blister chambers (alveoli; d, e, f), and the interstitial spaces (c’, g, h), as well as recording the topography of the alveoli thereon and determining the spatial relationships. The dimensions of the blister chambers were always measured at the base of the alveolus

For all analyses, we used the blister cards of the largest package size available in the market, which was aimed for long-term therapy (~ 100 d) and occasionally, if no such package was available, for a treatment duration of 30 d. By examining the largest marketed package size, this study evaluated the presumed most efficient packaging with the least amount of (primary and secondary) packaging material per dosage form and the least likelihood of empty areas without blisters (Fig. 1), resulting in a conservative estimate of waste production.

In the course of data collection, the same parameters were surveyed for all selected brands and collected in a Microsoft Excel file (Excel Version 16.62, Microsoft, Redmond, WA, USA). In addition to general conditions such as the number of dosage forms or the number of blister cards per pack, further measurements were performed (Fig. 1), which can be divided into 4 subanalyses:

1. Blister cards (weight (incl. dosage forms), length (i in Fig. 1), width (2 × b), and height)
2. Blister chamber (alveolus) (form (elongated/round), diameter (d) (if round) or length (f) and width (e) (if elongated or capsule), and largest (g) and smallest distance (h) between two blister chambers)
3. Dosage form (form, diameter (if round) or length and width (if elongated/capsule), height, and mass), and (if available)
4. Single blister compartments (number of large and small compartments per blister card), length (largest (a) and smallest (c)), width (b), and number and area (b × c’) of compartments without alveolus (empty blister field)

All weight measurements were taken with a digital scale (Sartorius Entris, Sartorius, Göttingen, Germany) accurate to the mg, or extracted from the drug information system AiD*Klinik*^®^ (Dosing GmbH, Heidelberg, Germany), and all linear measurements were performed with a digital caliper gauge (Carbon Fiber Composites Digital Caliper, Apfelkiste, Zeiningen AG, Switzerland) that allowed for a measurement accuracy of one mm.

### Quality assurance of data acquisition

Before the actual data collection was started, measurement accuracy and consistency of the investigator (OF-W) was tested in a pre-test after a learning phase and compared with the measurements of an experienced pharmacist (ADF). Based on this pre-test containing 15 SODF, which revealed complete agreement for all categorical assessments (Cohen’s kappa value 1.0) and almost complete agreement (97%) between both investigators in numerical data (222 measurements), it was decided that a single measurement per category and substance, carried out by one person was reliable.

### Calculation of ideal blister card dimensions

Because the distances between individual alveoli (g and h in Fig. 1) and between the alveolus and the edge of the blister card varied considerably, we also calculated the minimum dimensions of the blister card, assuming that the SODF were grouped into 2 rows, that the current alveolus dimensions remained unchanged, and that the distances between the SODF and to the edge were uniformly either 2, 3, or 4 mm (Eq. 1 for round SODF and Eq. 2 for elongated SODF).

1 $$\mathrm{Blister \;card\; area }= (2 \;d + 3 \;k) \times (k + 0.5\; n \;(k + d))$$

2 $$\mathrm{Blister\; card\; area }= (2 \;f + 3 \;k) \times (k + 0.5 \;n \;(k + e))$$

where *d* is the diameter of round alveoli, *f* the length, *e* the width of elongated alveoli, *k* is the distance between the alveoli, and *n* is the number of SODF on a blister card (see also Fig. 1).

To calculate the savings in surface area, the blister area measured in practice was taken as 100% and then the area of the optimized blister card was compared to the original area of the marketed product. The difference between these two values then corresponds to the savings in surface area and material.

### Estimation of blister card waste of solid oral dosage forms of the German pharmaceutical market

To be independent of the different package sizes prescribed, actual waste quantities of the best-selling 45 products (Table 2) were estimated based on the number of defined daily doses (DDD) of the corresponding brands dispensed in 2021 (Supplemental Table S1). The number of SODF consumed yearly was calculated by dividing the number of DDD prescribed [15] by the strength that was analysed in this study. This number was then divided by the number of dosage forms contained in the largest marketed package size as assessed in this study, giving the equivalents of drug packages. With these results and using the information on the number of SODF per blister card, the number of blister cards was calculated. These results were then multiplied by the weight of a blister card, giving the mass of waste per year in Germany for each preparation studied.

Assuming that their primary packaging characteristics are comparable to the observations in the group of the 45 most prescribed products, an extrapolation to the total market of reimbursable SODF was carried out. For this purpose, drug groups with mainly (> 50%) topically (dermatics, ophthalmics, and inhalatives) or parenterally administered medicines (e.g., immunostimulants, blood products, and allergens) were excluded. For the remaining drugs, we extracted the DDD reimbursed by the statutory health insurance funds in 2020 [15]. Using a rule of three, we extrapolated the amount of waste, assuming that the average amount of waste per DDD of the 45 best-selling products is also representative for the remaining preparations. Since, in addition, about 10.5% of the German population is privately insured [15], the result of the rule of three was multiplied by 1.105, which then gives the value for blister packaging waste for SODF of the entire German pharmaceutical market.

### Statistics

#### Descriptive statistics

Data are expressed as means ± standard deviation and range using Microsoft Excel. To account for possible uneven distribution of values, the relationship between variables was analyzed using Spearman correlation (Prism 9, Version 9.4.1., GraphPad Software Inc., San Diego, CA, USA). A *p* value < 0.05 was considered significant.

## Results

### Blister and dosage form characteristics

Of the 45 top-selling brands (Supplemental Table S1), 60% were round tablets, just over $\nicefrac{1}{3}$ were elongated tablets, and only 2 (4.4%) were capsules (Table 1). Depending on the potency of the active ingredient contained, the strengths of the SODF varied greatly (0.05–850 mg). On average, the volume of elongated tablets was more than twice that of round tablets, highlighting the considerable range (44.9–968 mm^3^) of dosage form volumes. As expected, due to the legal framework conditions, the pack size only fluctuated within very narrow limits. In contrast, the number of SODF on a blister card and thus the number of blister cards per package differed considerably. Even more variable was the total blister area with the largest blister card being 8.5 times larger than the smallest blister card. On average, round SODF had the smallest blister cards and capsules the largest. This also applies to the averaged area of the blister card per SODF (Table 1).

**Table 1:** Properties of the 45 most frequently prescribed solid oral dosage forms in Germany in 2020 whose primary packaging was an alveolus

**Characteristic**	**All**	**Round tablet**	**Elongated tablet**	**Capsule**^a^
Prescription medicines/pharmacy-only medicines (*n*)	45/0	27/0	16/0	2/0
Strength of SODF (mg), (mean ± SD; range)	103 ± 205 (0.05–850)	50.0 ± 109 (0.05–500)	203 ± 293 (5–850)	0.4/50
Package size (*n* SODF/package), (mean ± SD; range)	97.6 ± 27.2 (20–200)	97.9 ± 25.9 (50–200)	96.9 ± 31.8 (20–180)	100/100
Blister cards per package (*n*), (mean ± SD; range)	7.06 ± 2.97 (4–14)	6.62 ± 2.99 (4–14)	7.44 ± 2.97 (4–10)	10/10
SODF per blister card (n), (mean ± SD; range)	16.0 ± 6.86 (5–25)	17.4 ± 7.31 (5–25)	14.4 ± 6.02 (10–25)	10/10
Total blister card area (cm^2^), (2*b* × *i*)(mean ± SD; range)	373 ± 173 (95.4–811)	326 ± 120 (191–563)	440 ± 228 (95.4–811)	400/552
Blister card area used for SODF (cm^2^), (mean ± SD, range)	3.92 ± 1.74 (1.87–10.3)	3.49 ± 1.69 (2.02–10.3)	4.54 ± 1.76 (1.87–7.06)	4.00 / 5.52
Blister chamber (alveolus) area (mm^2^), (round: *π* × (*d*/2)^2^, elongated: *e* × *f*), (mean ± SD, range)	121 ± 50.9 (50.3–238)	100 ± 46.1 (50.3–227)	158 ± 65.5 (59.5–238)	115/115
Alveolus area percentage of total area (%), (mean ± SD, range)	31.2 ± 7.84 (15.1–49.1)	29.4 ± 7.65 (15.1–41.5)	35.1 ± 6.99 (18.6–49.1)	20.9/28.9
Volume of SODF (mm^3^), (mean ± SD; range)	249 ± 242 (44.9–968)	177 ± 132 (44.9–631)	365 ± 343 (68.6–968)	294/305
Alveolus area/volume of SODF (cm^2^/mm^3^), (mean ± SD; range)	0.79 ± 0.68 (0.22–3.43)	0.81 ± 0.71 (0.24–3.43)	0.82 ± 0.67 (0.22–2.04)	0.38/0.39
Strength/volume of SODF (mg/mm^3^), (mean ± SD; range)	0.25 ± 0.39 (0.0004–2.21)	0.22 ± 0.45 (0.0004–2.21)	0.33 ± 0.24 (0.03–0.88)	0.001/0.16
Weight of SODF (g), (mean ± SD; range)	0.24 ± 0.23 (0.04–0.94)	0.18 ± 0.12 (0.04–0.59)	0.36 ± 0.33 (0.08–0.94)	0.14/0.23
Total blister card weight (g), (mean ± SD; range)	14.2 ± 6.67 (3.20–34.6)	13.0 ± 5.98 (7.86–34.6)	15.6 ± 7.72 (3.20–33.1)	17.7/21.4
Tare weight percentage of packaging (%), (mean ± SD; range)	43.6 ± 14.7 (16.4–74.7)	45.8 ± 12.4 (23.6–74.7)	38.9 ± 17.7 (16.4–69.9)	43.2/60.8

The variables used for calculation of the respective data are specified in Fig. 1, and the corresponding equations are given in braces {}

*SODF* single oral dosage form

^a^Due to the small sample size (*n* = 2), only the measured values for the two capsules are mentioned in each case without calculating mean values

The corresponding characteristics of the 8 groups of generics are specified in Supplemental Table S3.

The analysis of the distances between the alveoli revealed significant differences between the individual products; the distances between blisters in the same row (h in Fig. 1) were on average 4.64 mm (range: 2–8 mm), in the same column (g in Fig. 1) 10.8 mm (range: 3–54 mm).

**Fig. 2:**
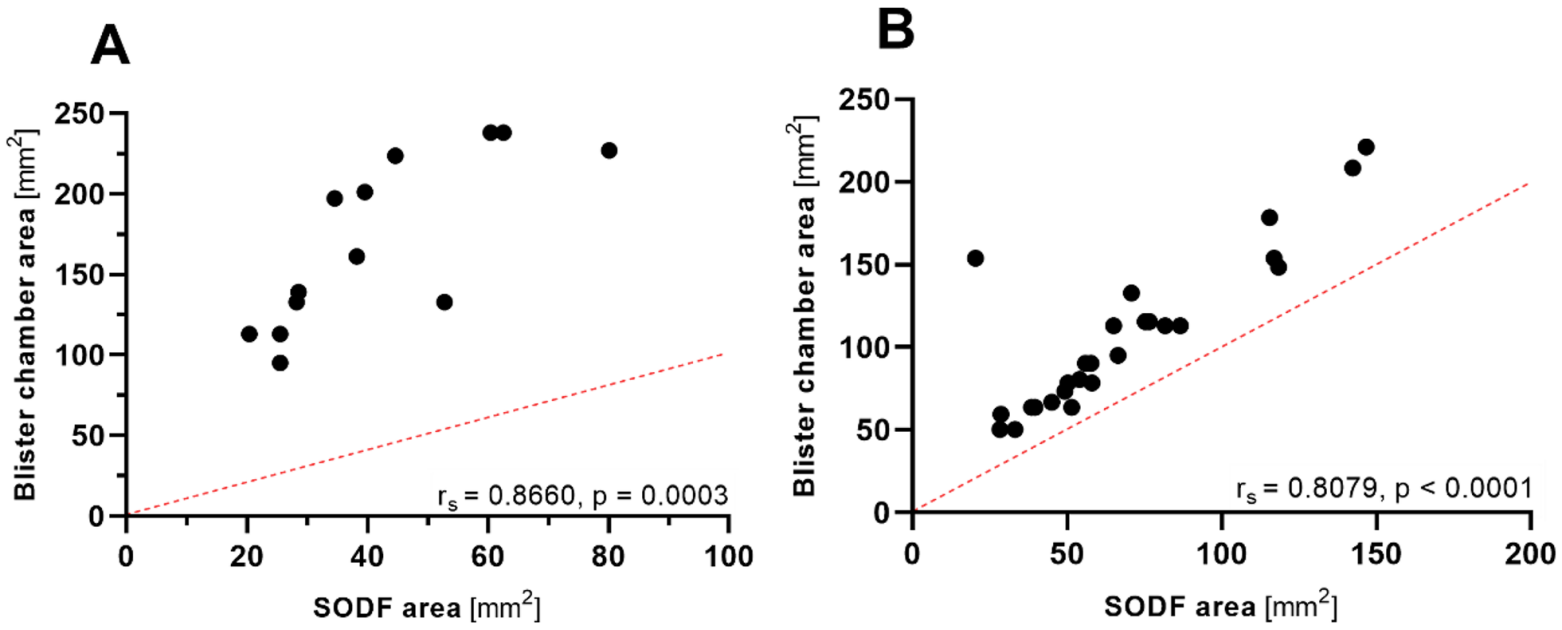
Relationship (Spearman rank correlation) between the areas of the solid oral dosage forms (SODF) and the respective blister chamber for the 45 best-selling brands. **A** The 13 SODF that were packaged in an aluminum-aluminum blister are shown on the left. **B** The remaining SODF were packaged in aluminum-plastic (*n* = 31) or pure plastic (*n* = 1) blisters and shown on the right. The red dashed lines show the lines of identity

An analysis of the relationship between the area covered by individual SODF and the size of the respective alveoli revealed considerable differences between different blister materials. The areas of the 13 aluminum-aluminum alveoli (mean ± SD: 170 mm^2 ^± 52.4) were 4.3 times larger than the SODF packed in them (41.6 mm^2^ ± 17.8), while for the group of mixed aluminum-plastic and pure plastic blisters, the average area of the packed SODF was 63.6 mm^2 ^± 32.8 and the corresponding alveolus area was 98.1 mm^2 ^± 46.0, i.e., only 1.7 times the area of the SODF. For both blister types, the individual alveolus area and the SODF area correlated strongly with each other (Fig. 2).

### Estimated annual production of packaging waste

In estimating the annual waste generation for the packaging of the 45 drugs most frequently prescribed in Germany, it was assumed that all blister cards of a brand are identical in the different package sizes available on the market. To be independent of the different package sizes prescribed, actual waste quantities (Table 2) were estimated based on the number of defined daily doses (DDD) of the corresponding brands dispensed in 2021 (SupplementalTable S1).

**Table 2:** Extrapolation of the blister packaging waste generated annually on the German pharmaceutical market

		Market covered by statutory health insurance	Entire market including private health insurance
TOP 45 preparations	Waste mass (t)	3500 t	3868 t
	Corresponding goods train length (m)	770 m	851 m
	Waste area^a^ (km^2^)	9.4 km^2^	10.4 km^2^
Entire market of preparations usually packaged in blisters	Waste mass (t)	7722 t	8533 t
	Corresponding goods train length (m)	1700 m	1880 m
	Waste area^a^ (m^2^)	19.6 km^2^	21.6 km^2^
	Annual waste area^a^ per inhabitant (m^2^)^b^		0.26

^a^The theoretical area covered by the packaging waste was calculated as the length of the blister cards (i in Fig. 1) × width of blister rows (number of blister rows x b in Fig. 1)

^b^Assuming a population size of 83.2 million inhabitants in Germany in 2021

The total annual amount of waste from blister packages of the 45 medicinal products for the patients with statutory health insurance amounted to 3500 t of composite waste (3868 t, if the 10.5% privately insured patients are added) consisting of aluminum, plastic material, and adhesives. Assuming that the specific gravity of blisters is 1.4 (polyvinyl chloride) and that the resulting waste is compacted to the maximum, the waste volume will result in 2500 m^3^ of composite waste, i.e., a cube with an edge length of 13.5 m. A goods train of approximately 770-m length would be required to transport only this waste of the 45 top-selling drugs from the patients with statutory health insurance (assuming that a 70-t freight wagon has a length of 15.4 m) (Table 2).

In a next step, we attempted to roughly estimate the total amount of blister waste for SODF in the German pharmaceutical market. For this purpose, the total number of DDD reimbursed by the statutory health insurance funds in 2020 was restricted to the substance groups consisting mainly of SODF [15]. Assuming that their primary packaging characteristics are comparable to the observations in the group of the TOP 45 most prescribed products, an extrapolation to the total market of reimbursable SODF was carried out (Table 2). In relation to the number of inhabitants in Germany, the total estimated annual waste produced for blister-packaging SODF in Germany was 0.26 m^2^.

### Variability between generics with the same active ingredient in the same strength

There were large differences between the different brands of a given generic drug (Supplemental Table S3). As an example, the weights of the SODF within one generic group differed by a factor of 1.02 (valproic acid) to 2.4 (acetylsalicylic acid) and SODF volumes by a factor of 1.06 (valproic acid) to 3.48 (acetylsalicylic acid). The mean blister card areas used for packaging a SODF of identical strength differed by a factor of 1.22 (valproic acid) to 4.12 (pantoprazole). Similarly, the percentage of packaging (tare weight) to total weight also varied widely between and within generic groups, with relative packaging weight lowest for an ibuprofen product (19.5%) and highest for a drug containing tamsulosin (63.4%).

### Relationship between the dimensions of the SODF and the dimensions of blister chambers and blister cards

**Fig. 3:**
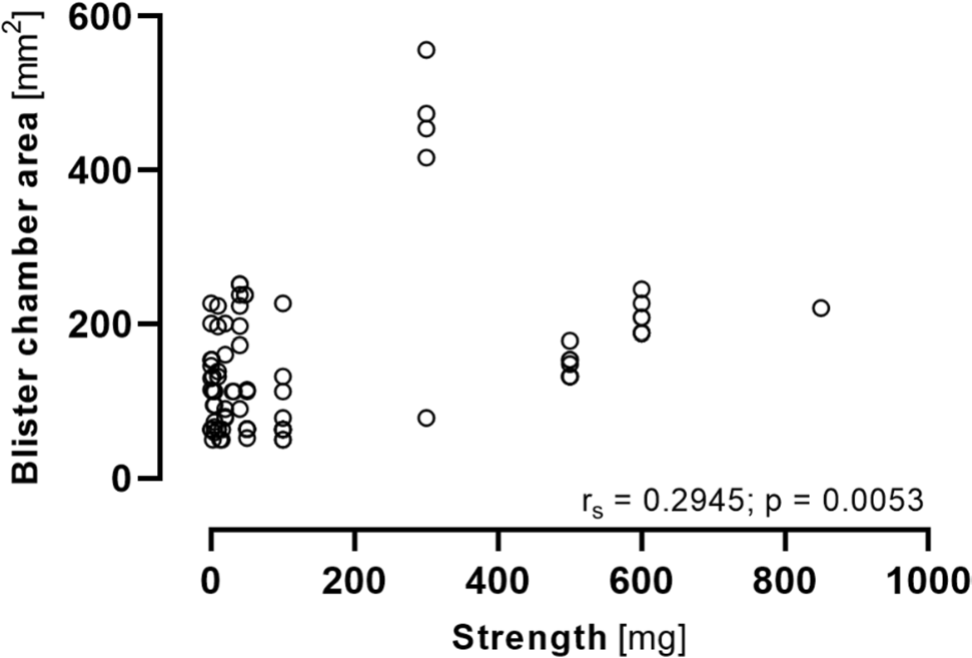
Relationship between the strength of the 88 solid oral dosage forms and occupied blister chamber areas (Spearman rank correlation)

To evaluate the relationship between SODF and packaging, we pooled the data of all analyzed brands, i.e., the 45 top-selling products (Supplemental Table S1) and 44 SODF of 8 groups of generic compounds (Supplemental Table S2), resulting in 88 SODF packaged in blisters (one brand was included in both groups and was therefore only considered once). On average, the blister chambers (alveoli) took up $\nicefrac{1}{4}$ – $\nicefrac{1}{3}$ of the blister card’s surface area and thus also of its weight, while $\nicefrac{2}{3}$ were occupied by gaps, welds, or empty areas without blisters, and in all instances accounted for more than half of the area of the blister cards. There was a weak correlation between dosage strength and the area of the blister chamber (Fig. 3) but there was no correlation between strength and SODF volume or between SODF volume and blister chamber area or blister gross weight. Large variability was also found in the weight ratio between blister cards and packaged SODF, resulting in a percentage of the packaging’s own weight (tare weight) ranging from 16–75% of the total weight.

### Projections for optimising the demand for packaging material based on dosage forms

The average area of a blister chamber (alveolus) of the 45 most frequently prescribed brands was 144 mm^2^ (Table 1), which was significantly (206%) larger than the area covered by the oral dosage form, suggesting that space around the dosage form is rather variable. Even more variable was the interspace between blister chambers with smallest distances being on average 4.7 mm between two blisters but ranging from 2 to 9 mm.

The average area of the blister cards of elongated tablets and capsules was 57.1 cm^2^ ± 19.4 and 52.1 cm^2^ ± 13.0 for the round tablets. Assuming that the minimum distance observed between two blister chambers (2 mm) is sufficiently large to securely seal all blister chambers, the optimised blister card area (Eqs. 1 and 2) was 39–40% smaller than the area of the currently marketed drug products of the 45 most frequently prescribed brands (Table 3), if there is only one blister row. If the blisters are arranged in two rows, as it is usually the case, and the blister spacing of 2 mm would be maintained, the optimized blister card area was even 42% smaller than the area of the currently marketed drug products. Assuming that all aluminum-aluminum blister cards require a minimum alveolus spacing of up to 4 mm and all other blister cards (plastic/aluminum, or only plastic) require a spacing of 2 mm and that it is a standard blister card with two blister rows, the achieved savings would be 37%.

**Table 3:** Blister card areas of the most sold 18 elongated or oval tablets and capsules and the most sold 27 round tablets before and after optimization

SODF	Area (cm^2^)	Alveolus distance (mm)	Optimized alveolus area [cm^2^],		
	Mean (± standard deviation) (defined as 100%)		Mean (± standard deviation) % savings in surface area		
			Blister chamber distance		
			2 mm	3 mm	4 mm
Elongated tablets and capsules (*n* = 18)	57.1 (± 19.4) 100%	5.3 (± 1.7)	33.3 (± 14.5) 42%	39.2 (± 16.4) 32%	45.7 (± 18.4) 20%
Round tablets (*n* = 27)	52.1 (± 13.0) 100%	4.3 (± 1.5)	30.3 (± 11.2) 42%	35.6 (± 13.2) 32%	43.3 (± 15.4) 17%

*SODF* single oral dosage form

Assuming that for technical reasons a minimum welding area of 4 mm size is required for aluminum-aluminum blisters and 2 mm for all other blister materials, the blister card area saved for the 45 best-selling SODF in Germany would be 37% (Table 4).

**Table 4:** Blister card areas (blister chambers arranged in two rows) before and after optimization including different interspaces depending on blister composition (aluminum-aluminum blister = 4 mm; all others = 2 mm)

Marketed product, mean (± standard deviation) (defined as 100%)		Optimised blister chamber area (cm^2^), mean (± standard deviation); % savings in surface area	
Area (cm^2^)		Area (cm^2^)	
Round tablets	Elongated tablets	Round tablets	Elongated tablets
52.1 (± 13.0); 100%	57.1 (± 19.4); 100%	33.4 (± 14.7); 37%	37.4 (± 18.6); 37%

We then examined how the arrangement of the blisters and the spacing between the blisters affected the total area of the blister cards. Therefore, for the 27 round SODF present among the 45 best-selling brands in Germany, we calculated the areas required to arrange 24 blister chambers in 1, 2, 3, or 4 rows (Table 5). The closer the arrangement was to a square, the smaller the space requirement was, and from alveolus spacings of 3 mm, the composite material for the blister chamber interspaces exceeded the packaging material requirement of the blister chambers.

**Table 5:** Impact of the blister arrangement and blister interspace on the blister area required for 24 round solid oral dosage forms

Blister arrangement [row × n SODF]	Minimum distance between 2 blister chambers			
	2 mm	3 mm	4 mm	5 mm
	Theoretical area of blister interspace (cm^2^ (% of 4 × 6 arrangement)^a^)			
1 × 24	47.1 (109%)	57.6 (112%)	69.2 (115%)	81.7 (117%)
2 × 12	44.2 (102%)	53.0 (103%)	62.6 (104%)	72.9 (105%)
3 × 8	43.5 (101%)	51.8 (101%)	60.8 (101%)	70.5 (101%)
4 × 6 (defined as 100%)	43.2 (100%)	51.3 (100%)	60.2 (100%)	69.7 (100%)

^a^Percentage values are normalized to the 4 × 6 blister arrangement of the corresponding column, which was set to 100%

## Discussion

While climate change is responsible for a significant proportion of morbidity and excess mortality by non-optimal temperatures [16], the health care system itself is a major driver of climate change. With an estimated 55 million tons of CO_2_ emitted in 2014, Germany’s health care system ranked fifth after China, the USA, Japan, and India, and the health carbon footprint amounted to almost 7% of the national carbon footprint [8]. Direct emissions of health care facilities (Scope 1) and emissions from direct energy purchases (Scope 2) contribute to only a minority of emissions, while greenhouse gases resulting from services and the production of goods (such as drugs and packaging) for health care (Scope 3) account for more than 80% [17].

In Germany, more than 1.5 bn medication packages are prescribed every year [10], most of which are SODF packaged in aluminum-plastic blisters [1]. However, it seems that little effort is made to minimise the waste of these disposable products, which are usually made of composite materials that currently have limited recyclability. Our study confirms that there is considerable room for improvement in the medical packaging industry as far as sustainability is concerned. Different aspects could be improved, such as type of packaging material chosen, recycling of waste material, as well as the dimensions of the primary and secondary packaging.

This study has shown that significant amounts of packaging material can be saved simply by choosing the dimensions of primary packaging wisely and changing 2 variables: First and foremost, efforts should be made to minimize the distance between alveoli to a technical minimum, i.e., 2 mm for thermoformed composite material and 4 mm for pure aluminum blisters. This measure alone could already save 37% of primary packaging material. Secondly, the spatial arrangement can be optimized by choosing a square instead of a rectangular arrangement, which leads to further savings of several percent. In terms of the total current packaging waste for SODF, this would correspond to an annual saving of more than 3300 t of composite-material waste in Germany alone.

Nevertheless, this estimate for the entire market should be treated with caution, because a number of assumptions had to be made (all prescriptions as 3-month packages, generalisation of the primary packaging requirements to all DDD of a certain indication group, exclusion of certain indication categories). It should also be considered that galenic formulations other than SODF may be packaged in blisters (e.g., suppositories, capsules for inhalation, powder for inhalation, and ointments) for which similar optimization rules would apply.

While pure aluminium blisters are often chosen for reasons of product protection, from an ecological perspective, they have several disadvantages: (i) aluminum-aluminum blisters usually have both larger blister chambers and larger blister interspaces, which in combination lead to larger and often very large blister cards, (ii) the production of aluminum-aluminum blisters is much more energy-intensive than thermoformed blisters, and (iii) because its specific weight is about twice that of polyvinyl chloride or polyethylene, transportation cost can contribute significantly to the environmental impact of a medicine packaged in aluminum-aluminum blisters [12]. This leads to a waste of two resources: aluminum and energy. On the other hand, aluminum is recyclable [18] suggesting that pure aluminum blisters should only be produced if it can be guaranteed that significant fractions of the aluminum will be consequently recycled. Although the recycling of these cold-formed blisters would in principle be much easier than that of the complex thermoformed blisters made of laminates, not even the valuable aluminum is recycled today.

Available alternatives to blister packaging are multidose containers, the ecological footprint of which is smaller [13]. In Germany, multidose containers are rare, and only two such products were found among the 50 most frequently prescribed medicines. The environmental performance of multidose containers may be better than that of blister packs [13], but they may have disadvantages in terms of protecting sensitive SODF from environmental effects and handling the container [1], as opening the container may be difficult, especially for older patients [19–21]. However, also blister packs are challenging to handle, and older patients often report that they are unable to push SODF through blister foils or remove the SODF from the card intact [20, 22], especially if the SODF is significantly smaller than the alveolus [23]. The smallest distance between two alveoli found in blister cards on the German market was 2 mm. To our knowledge, the influence of the spacing between two SODF on the success of extraction of their contents has not been studied in detail. However, in surveys of geriatric populations, the distance between individual blisters was also occasionally mentioned as a potential problem [19], but because this was grouped into a small varia group with a wide range of challenges, the exact frequency is unknown and likely low.

Finally, a significant proportion of medicines (15%, [24]) is only manufactured and not consumed, which is also a problem that should be addressed nowadays, if the content is not needed or patient adherence is low. In such cases even the most efficient and sustainable packaging is worthless, and the product adds to the waste disposal issue with not only the packaging but also the actual left-over medication, which, due to the active ingredient, might even be a hazardous substance for the environment.

Furthermore, all packaging, regardless of whether its contents are used or not, has to be disposed of sooner or later and hardly anything is recycled [25]. Blister cards have been identified as a packaging strategy with considerable potential for optimisation [12]. In addition to reducing the number of unused medicines (and their packaging) going into waste, it would therefore be all the more important to reduce packaging material in the environment. This could be done by reducing unnecessarily large blister cards (as shown in this study), by increasing recycling efforts, and by developing new packaging materials and strategies. The latter could include (i) biodegradable packaging, (ii) recyclable packaging with a shelf life that is not multiple times longer than the packaged contents, or (iii) reusable primary packaging material [26].

While it is understandable that pharmaceutical companies will not change blister design without necessity due to regulatory challenges and costs, it could be different for generic drugs. Because a large amount of data is usually available for generics, including the long-term stability of the originator product, generic manufacturers would have the opportunity to optimize their blister design before applying for marketing authorisation, taking into account the existing stability data and possible options to minimize foil consumption. We therefore also assessed the dimensions of generics of the same strength. This showed that the standard deviations of the means and the ranges were often smaller for generics compared with the 45 SODF with the highest sales. This suggests certain regularities within the same group of active ingredients and strengths (and possibly additives). The differences between the individual brands of metformin, sertraline, and valproic acid were particularly small, indicating that it would in principle be possible to use consistent blisters for all of these brands. On the other hand, in individual cases, the heterogeneity was surprisingly large and unexplained. For example, in the pantoprazole 40 mg generics, which were exclusively tablets, the area occupied per blister differed more than fourfold between brands, while the volumes of the dosage forms differed only 1.3-fold. Whether in these cases it was cheaper to stick to already existing tools already in place (forming plates and stamps and perforating and cutting tools) than to reduce packaging waste is not known.

## Strengths and limitations

A strength of this work is that a large fraction of the most often prescribed medicines in Germany were analysed (alone the TOP 45 preparations analysed correspond to about 45% of all DDD prescribed annually in Germany) and that also a considerable number of generics of identical strengths were compared. Other strengths of this study are that several different packaging materials were included in the review and numerous different characteristics of the blister packaging, such as size, volume, and mass were studied as well as extensive quality assurance measures were taken for data acquisition to generate reproducible and reliable data.

Despite these important strengths, there are also a number of limitations worth mentioning. First, the rounded blister card corners were omitted from the calculation of the blister card area. However, this does not matter or is even more correct than if the blister area with rounded corners had been included in the calculation, as the blisters are punched, and the blister remnants of the corners end up in the waste anyway. Second, while it can be said that a large and significant number of preparations have been analysed, this nevertheless represents only a fraction of the very large German pharmaceutical market and, therefore, the results cannot be extrapolated to the entire market with complete certainty. In this context, it is also important to note that our results (other than for the European pharmaceutical market) are certainly not transferable to the pharmaceutical market in the USA, since blister cards are rare there. Third, the micro-climate in the individual alveoli may require larger alveoli for SODF stability reasons [27]; no attempt was made to optimize blister chamber volumes. Finally, we have not found any objective reasons (e.g., standards of the regulatory authorities, regimentations of the warehouses in the pharmacies (e.g., drawer size), or the commissioning robots) for the current dimensions of the individual blister cards, and they are certainly not exclusively due to the SODF packed with them. It is conceivable that logistical aspects of these goods, which are often transported over long distances (incl. overseas in containers), could have an influence on blister cards and thus package size. Whatever the case, it must be clarified in the very near future how these packaging waste amounts can be reduced. A first and immediately possible step would be to optimise blister card dimensions and to ensure their recycling as an additional measure, even if this is more labor-intensive and thus less economical than simply discarding the material.

## Conclusion

In summary, every blister pack produced to package SODF must ultimately be disposed of, which in the case of the German pharmaceutical market is an estimated mass of 8533 t of composite materials. Our study revealed that the simple strategy of minimizing the distance between individual blister chambers could immediately and substantially improve this situation and reduce waste from these difficult-to-recycle composite packaging materials by well over one-third.

### Supplementary Information

Below is the link to the electronic supplementary material.

Supplementary file1 (DOCX 42 KB)

## Data Availability

The datasets generated during and/or analyzed during the current study are available on request from the corresponding author.
