# Supplementary material for: Untapped options to reduce waste from blister packaging for tablets and capsules
Source: Eur J Clin Pharmacol. 2023 Nov 18;80(1):151–61. doi: 10.1007/s00228-023-03594-1 (PMC10781798; doi:10.1007/s00228-023-03594-1)
Supplement: Supplementary file 1 — Supplementary file1 (DOCX 42 KB) [file 228_2023_3594_MOESM1_ESM.docx]

# Supplemental Material

# Eur J Clin Pharmacology

**Untapped options to reduce waste from blister packaging for tablets and capsules**

Olivia C. Falconnier-Williams^1^, Walter Taeschner^2^, Andreas Hille^3^, Ariane D. Falconnier^4^, Walter E. Haefeli^1*^

^1^ Department of Clinical Pharmacology and Pharmacoepidemiology, Heidelberg University Hospital, Im Neuenheimer Feld 410, 69120 Heidelberg, Germany

^2^ Frosch Apotheke, Basler Str. 19, 79539 Lörrach, Germany

^3^ Drossapharm AG, Pharmaceuticals, Birsweg 1, 4144 Arlesheim, Switzerland

^4^ 4104 Oberwil, Switzerland

**Supplemental Table S1**: The top-selling 45 drugs with solid oral dosage forms whose primary packaging in the largest marketed package size was a blister pack.

| **Rank** | **Brand name** | **Active ingredient** | **Strength [mg]** | **DDD [mg]** | **DDD dispensed in Germany in 2021 [n]** | **Dosage form** | **Blister composition** |
| --- | --- | --- | --- | --- | --- | --- | --- |
| 1 | Novaminsulfon Lichtenstein | Metamizole | 500 | 3000 | 209,213,500 | Elongated | Al / plastic |
| 2 | Ibuflam/-Lysin | Ibuprofen | 600 | 1200 | 413,851,100 | Elongated | Al / plastic |
| 3 | RamiLich | Ramipril | 5 | 2.5 | 2,725,245,500 | Elongated | Al / plastic |
| 4 | Torasemid AL | Torasemide | 10 | 15 | 677,745,200 | Round | Al / plastic |
| 5 | L-Thyroxin Henning | L-thyroxine | 0.1 | 0.15 | 537,000,500 | Round | Al / plastic |
| 6 | Amlodipin Dexcel | Amlodipine | 10 | 5 | 958,699,800 | Round | Al / plastic |
| 7 | Bisoprolol-ratiopharm | Bisoprolol | 1.25 | 10 | 319,286,300 | Round | Al / plastic |
| 8 | MetoHEXAL/ MetoHEXAL succ | Metoprolol | 100 | 150 | 317,215,600 | Octagonal^‡^ | Al / plastic |
| 9 | L-Thyrox HEXAL | L-thyroxine | 0.05 | 0.15 | 372,371,600 | Round | Al / plastic |
| 10 | Metformin Lich | Metformin | 850 | 2000 | 393,648,800 | Elongated | Al / plastic |
| 11 | Panto/ Pantoprazol Aristo | Pantoprazole | 40 | 40 | 677,067,100 | Oval | Plastic |
| 12 | Ramipril-1 A Pharma | Ramipril | 10 | 2.5 | 1,083,729,600 | Elongated | Al / Al |
| 13 | Tilidin AL comp | Tilidine | 100 | 200 | 148,071,800 | Round | Al / Al |
| 14 | Simva BASICS | Simvastatin | 20 | 30 | 438,055,900 | Elongated | Al / plastic |
| 15 | Eliquis | Apixaban | 2.5 | 10 | 309,292,200 | Round | Al / plastic |
| 16 | Lercanidipin Omniapharm | Lercanidipine | 20 | 10 | 536,280,000 | Round | Al / plastic |
| 17 | BisoHEXAL | Bisoprolol | 2.5 | 10 | 171,529,800 | Round | Al / Al |
| 18 | Bisoprolol-1 A Pharma | Bisoprolol | 5 | 10 | 163,990,500 | Round | Al / Al |
| 19 | Simva Aristo | Simvastatin | 20 | 30 | 345,598,700 | Elongated | Al / plastic |
| 20 | Metoprolol/-succ-1 A Pharma | Metoprolol | 47.5 | 143* | 181,629,400 | Elongated | Al / Al |
| 21 | Allopurinol AL | Allopurinol | 300 | 400 | 184,573,500 | Round | Al / plastic |
| 22 | Candesartan-1 A Pharma | Candesartan | 4 | 8 | 629,862,200 | Round | Al / Al |
| 23 | Novaminsulfon-ratiopharm | Metamizole | 500 | 3000 | 33,551,200 | Round | Al / plastic |
| 24 | Euthyrox | L-thyroxine | 0.075 | 0.15 | 197,879,000 | Round | Al / plastic |
| 25 | Atorvastatin Accord | Atorvastatin | 20 | 20 | 453,998,400 | Round | Al / Al |
| 26 | Ramipril AbZ | Ramipril | 5 | 2.5 | 668,462,100 | Elongated | Al / Al |
| 27 | Prednisolon acis | Prednisolone | 50 | 10 | 174,210,600 | Round | Al / plastic |
| 28 | Xarelto | Rivaroxaban | 15 | 20 | 244,967,400 | Round | Al / plastic |
| 29 | Candecor | Candesartan | 16 | 8 | 408,640,600 | Round | Al / plastic |
| 30 | Pantoprazol-1 A Pharma | Pantoprazole | 20 | 40 | 336,199,000 | Oval | Al / Al |
| 31 | Candesartan Zentiva | Candesartan | 32 | 8 | 412,547,900 | Round | Al / plastic |
| 32 | Pregabalin-neuraxpharm | Pregabalin | 50 | 300 | 65,391,000 | Capsule | Al / plastic |
| 33 | Metoprolol/-succ-ratiopharm | Metoprolol | 47.5 | 143* | 120,647,200 | Elongated | Al / Al |
| 34 | Torasemid-1 A Pharma | Torasemide | 2.5 | 15 | 194,331,200 | Round | Al / Al |
| 35 | Enalapril AL | Enalapril | 10 | 10 | 263,808,600 | Round | Al / Al |
| 36 | Amoxi-1 A Pharma | Amoxicillin | 500 | 1500 | 23,771,700 | Elongated | Al / plastic |
| 37 | Tamsulosin Zentiva | Tamsulosin | 0.4 | 0.4 | 222,011,300 | Capsule | Al / plastic |
| 38 | Amlodipin-1 A Pharma | Amlodipine | 5 | 5 | 271,699,200 | Oval | Al / plastic |
| 39 | Amlodipin Fair-Med | Amlodipine | 5 | 5 | 266,598,500 | Round | Al / plastic |
| 40 | HCT Dexcel | HCT | 12.5 | 25 | 160,724,700 | Round | Al / plastic |
| 41 | Thyronajod | L-thyroxine + KI | 1 | 1^†^ | 209,570,800 | Round | Al / plastic |
| 42 | RamiLich comp | Ramipril + HCT | 1 | 1^†^ | 203,722,500 | Oval | Al / plastic |
| 43 | Citalopram Aristo | Citalopram | 30 | 20 | 192,023,700 | Round | Al / plastic |
| 44 | Atorvastatin-ratiopharm | Atorvastatin | 10 | 20 | 292,826,000 | Oval | Al / Al |
| 45 | ASS AL TAH/-protect | Acetylsalicylic acid | 100 | 100 | 193,549,300 | Round | Al / plastic |

* The DDD of metoprolol tartrate (150) was adjusted to account for the succinate contained in this brand. ^†^ For combination products, the DDD was set to 1 SODF. ^‡^ Octagonal tablets were treated as round tablets.

Al: aluminum, DDD: defined daily doses, HCT: hydrochlorothiazide; KI: potassium iodide.

**Supplemental Table S2**: Solid oral dosage forms of generic competitors whose primary packaging was an alveolus.

| **Active ingredient** | **Strength [mg]** | **Tablet [N products]** | **Capsule [N products]** | **Brands [N]** | **Brands packaged in alveoli [N]** |
| --- | --- | --- | --- | --- | --- |
| Acetylsalicylic acid | 100 | 9 | 0 | 9 | 9 |
| Ibuprofen | 600 | 7 | 0 | 7 | 7 |
| Metformin | 500 | 5 | 0 | 5 | 5 |
| Omeprazole* | 40 | 0 | 5 | 5 | 0 |
| Pantoprazole* | 40 | 11 | 0 | 11 | 7 |
| Sertraline | 50 | 6 | 0 | 6 | 6 |
| Tamsulosin hydrochloride | 0.4 | 2 | 4 | 6 | 6 |
| Valproic acid* | 300 | 6 | 0 | 6 | 4 |
| All |  | 46 | 9 | 55** | 44 |

Always the largest marketed package size was evaluated.

* Products whose primary packaging was not a single blister pack (all products containing omeprazole, four containing pantoprazole, and 2 containing valproic acid were packaged in multi-dose containers).

** The 55 brands were produced by 21 different manufacturers.

**Supplemental Table S3**: Properties of the seven groups of frequently prescribed generics produced by 21 manufacturers and marketed as solid oral dosage forms in single blister packs.

|  | **All**  (n = 38)* | **Acetylsalicylic acid**  (n = 9) | **Ibuprofen**  (n = 7) | **Metformin**  (n = 5) | **Pantoprazole**  (n = 7)** | **Sertraline**  (n = 6) | **Tamsulosin HCl**  (n = 6) | **Valproic acid**  (n = 4)*** |
| --- | --- | --- | --- | --- | --- | --- | --- | --- |
| Prescription medicines / pharmacy-only medicines [n]  (numbers referring to blistered brands) | 35/9 | 0/9 | 7/0 | 5/0 | 7/0 | 6/0 | 6/0 | 4/0 |
| Dosage form [tablets/capsules] | 40/4 | 9/0 | 7/0 | 5/0 | 7/0 | 6/0 | 2/4 | 4/0 |
| Strength of SODF [mg]  (mean ± SD; range) | 188 ± 229 (0.04-600) | 100 ± 0.00 (100-100) | 600 ± 0.00 (600-600) | 500 ± 0.00 (500-500) | 40.0 ± 0.00 (40.0-40.0) | 50.0 ± 0.00 (50.0-50.0) | 0.40 ± 0.00 (0.40-0.40) | 300 ± 0.00 (300-300) |
| Package size [n dosage forms / package]  (mean ± SD; range) | 109 ± 25.7 (100-180) | 100 ± 0.00 (100-100) | 100 ± 0.00 (100-100) | 180 ± 0.00 (180-180) | 100 ± 0.00 (100-100) | 100 ± 0.00 (100-100) | 100 ± 0.00  (100-100) | 100 ± 0.00 (100-100) |
| Blister cards per package [n]  (mean ± SD; range) | 9.30 ± 3.03 (4.00-10.0) | 6.33 ± 2.78 (4.00-10.0) | 10.0 ± 0.00 (10.0-10.0) | 13.8 ±4.02 (9.00-18.0) | 8.29 ± 2.93 (4.00-10.0) | 10.0 ± 0.00 (10.0-10.0) | 9.17 ± 2.04 (5.00-10.0) | 10.0 ± 0.00 (10.0-10.0) |
| SODF per blister card [n]  (mean ± SD; range) | 13.1 ± 5.41 (10.0-25.0) | 18.3 ± 6.61 (10.0-25.0) | 10.0 ± 0.00 (10.0-10.0) | 14.0 ± 4.18 (10.0-20.0) | 14.3 ± 7.32 (10.0-25.0) | 10.0 ± 0.00 (10.0-10.0) | 11.7 ± 4.08 (10.0-20.0) | 10.0 ± 0.00 (10.0-10.0) |
| Total blister card area [cm^2^]  {2b × i} (mean ± SD; range) | 522 ± 226 (201-1008) | 262 ± 46.0 (201-333) | 562 ± 71.2 (513-665) | 759 ± 68.9 (648-816) | 563 ± 227 (221-910) | 363 ± 33.6 (319-418) | 512 ± 141  (352-665) | 921 ± 80.3 (829-1008) |
| Blister card area used for SODF [cm^2^]  (mean ± SD, range) | 4.84 ± 2.10 (2.02-10.1) | 2.62 ± 0.46 (2.02-3.33) | 5.62 ± 0.71 (5.13-6.65) | 4.22 ± 0.38 (3.60-4.54) | 5.63 ± 2.27 (2.21-9.10) | 3.63 ± 0.34 (3.19-4.18) | 5.12 ± 1.41 (3.52-6.65) | 9.21 ± 0.80 (8.29-10.1) |
| Blister chamber (alveolus) area [mm^2^]  {round: π × (d/2)^2^, elongated: e × f} (mean ± SD, range) | 169 ± 28.9  (50.3-556) | 67.8 ± 18.9  (50.3-113) | 205 ± 23.1  (189-245) | 137 ± 9.48  (133-154) | 204 ± 57.8  (90.3-252) | 62.3 ± 4.90  (52.3-64.3) | 169 ± 37.0  (130-227) | 475 ± 59.0  (417-556) |
| Alveolus area percentage of total area [%]  (mean ± SD, range) | 32.6 ± 10.3 (15.4-55.2) | 25.8 ± 4.50 (19.1-35.3) | 36.6 ± 1.38 (34.1-38.8) | 32.9 ± 5.65 (29.3-42.8) | 38.2 ± 7.84 (27.7-50.3) | 17.2 ± 1.20 (15.4-18.4) | 34.0 ± 6.77 (23.2-43.8) | 51.5 ± 2.64 (49.1-55.2) |
| Volume of SODF [mm^3^]  (mean ± SD; range) | 452 ± 338 (115-1478) | 192 ± 85.3 (115-400) | 1074 ± 275 (863-1478) | 596 ± 16.8 (582-623) | 246 ± 25.7 (210-272) | 145 ± 23.3 (124-187) | 392 ± 99.3  (286-517) | 555 ± 15.1 (544-578) |
| Alveolus area per volume of SODF [cm^2^/mm^3^]  (mean ± SD; range) | 0.47 ± 0.26 (0.15-1.14) | 0.38 ± 0.08 (0.28-0.55) | 0.20 ± 0.03 (0.15-0.22) | 0.23 ± 0.02 (0.21-0.26) | 0.83 ± 0.22 (0.40-1.14) | 0.44 ± 0.07 (0.34-0.52) | 0.46 ± 0.18 (0.30-0.70) | 0.86 ± 0.11 (0.77-1.01) |
| Strength / volume of SODF [mg/mm^3^]  (mean ± SD; range) | 0.43 ± 0.28 (0.0008-0.87) | 0.59 ± 0.18 (0.25-0.87) | 0.59 ± 0.12 (0.41-0.70) | 0.84 ± 0.02 (0.80-0.86) | 0.16 ± 0.02 (0.15-0.19) | 0.35 ± 0.05 (0.27-0.40) | 0.001 ± 0.0003 (0.0008-0.001) | 0.54 ± 0.01 (0.52-0.55) |
| Weight of dosage form [g]  (mean ± SD; range) | 0.38 ± 0.26 (0.15-1.04) | 0.20 ± 0.07 (0.15-0.36) | 0.88 ± 0.12 (0.72-1.04) | 0.55 ± 0.03 (0.52-0.59) | 0.22 ± 0.02 (0.20-0.24) | 0.16 ± 0.01 (0.15-0.19) | 0.27 ± 0.06 (0.19-0.34) | 0.45 ± 0.01 (0.45-0.46) |
| Total blister card weight [g]  (mean ± SD; range) | 2.28 ± 0.55  (1.11-3.81) | 2.14 ± 0.85  (1.11-3.81) | 2.52 ± 0.41  (2.07-2.96) | 2.30 ± 0.47  (1.81-2.89) | 2.33 ± 0.71  (1.45-3.44) | 1.59 ± 0.19  (1.41-1.86) | 2.37 ± 0.77  (1.77-3.43) | 2.95 ± 0.10  (2.87-3.08) |
| Tare weight percentage of packaging [%]  (mean ± SD; range) | 37.4 ± 10.9 (19.5-63.4) | 38.5 ± 5.21 (29.6-45.9) | 22.4 ± 3.01 (19.5-27.4) | 23.4 ± 2.41 (20.6-25.9) | 44.0 ± 6.12 (36.5-54.2) | 49.7 ± 3.36 (46.8-54.9) | 43.3 ± 10.5 (33.7-63.4) | 39.5 ± 1.01 (38.4-40.6) |

SODF: solid oral dosage form. The variables used for calculation of the respective data are specified in Figure 1 and the corresponding equations are given in braces {}. All 5 omeprazole preparations were packaged in multi-dose containers, which is why they are not included in this analysis.

* "All" refers to all the investigated prescribed generics that were packaged in blister cards and excludes those that were packaged in multi-dose containers.

** Four of the 11 pantoprazole preparations were packaged in multi-dose containers, which is why only the 7 preparations that were packaged in blister cards were considered for the results.

*** Two of the 6 valproic acid preparations were packaged in multi-dose containers, which is why only the 4 preparations that were packaged in blister cards were considered for the results.
